# Metanephric adenosarcoma: a rare case with immunohistochemistry and molecular analysis

**DOI:** 10.1186/s13000-014-0179-7

**Published:** 2014-09-30

**Authors:** Tiefen Su, Fei Yan, Pengcheng Zhu

**Affiliations:** Institute of Pathology, Tongji Hospital, Tongji Medical College, Huazhong University of Science and Technology, Jiefang Dadao, 1095, Wuhan, 430030 China; Department of Oncology, Zhongshan Hospital of Hubei Province, Zhongshan Dadao 26, Wuhan, 430000 China

**Keywords:** Metanephric adenosarcoma, Metanephric neoplaisa, Kidney

## Abstract

**Background:**

Metanephric neoplasms comprised a spectrum of kidney tumors containing renal epithelial or stromal cells or both, including metanephric adenoma, metanephric stromal tumor, and metanephric adenofibroma. The majority of cases were benign; only one case of “metanephric adenosarcoma” had been reported in the English literature.

**History:**

We present the case of a 69-year-old man who developed a neoplasm composed of renal epithelial component identical to metanephric adenoma combined with malignant spindle-cell stroma. The epithelial component was positive for CD57, AE1/AE3, but negative for WT-1, CD56, SYN, and CgA; whereas the sarcomatous component was negative for epithelial markers, SMA, Caldesmon, MyoD1, Myogenin, and S-100; and positive for vimentin, CD10, and WT1 focally. No specific sarcoma differentiation was apparent in the stroma by immunohistochemistry, and no SYT-SS18 rearrangement or BRAF mutation was detected by molecular analysis.

A diagnosis of metanephric adenosarcoma was made because of the morphological features and immunohitochemistry and molecular pathology analysis.

**Clinical significance:**

We believe that metanephric adenosarcoma should be in the expanded spectrum of metanephric neoplasia as a malignant stromal variant.

**Conclusions:**

We report a rare case of metanephric adenosarcoma with immunohistochemistry and molecular analysis and emphasize the histopathologic features and differential diagnosis of the rare lesion to promote a better and broader understanding of this less understood subject.

**Virtual Slides:**

The virtual slide(s) for this article can be found here: http://www.diagnosticpathology.diagnomx.eu/vs/13000_2014_179

## Background

Metanephric neoplasms, as uncommon renal tumors [1], have so far been reported to encompass metanephric adenoma (purely epithelial neoplasm), metanephric stromal tumor (purely stromal tumor), and metanephric adenofibroma (biphasic tumor). Most of these tumors have been known for their benign behavior. Before present report, only one case of “metanephric adenosarcoma” was described by M. M. Picken et al. as a metanephric tumor with malignant stroma [2]. Our case represents the second report of this lesion in the literature with immunohistochemistry and molecular pathologic analysis. The study was approved by the Ethics Committee of Tongji Hospital, Tongji Medical College, Huazhong University of Science and Technology, Wuhan, China. Written informed consent was obtained from the patient prior to death.

## Case report

**Figure 1:**
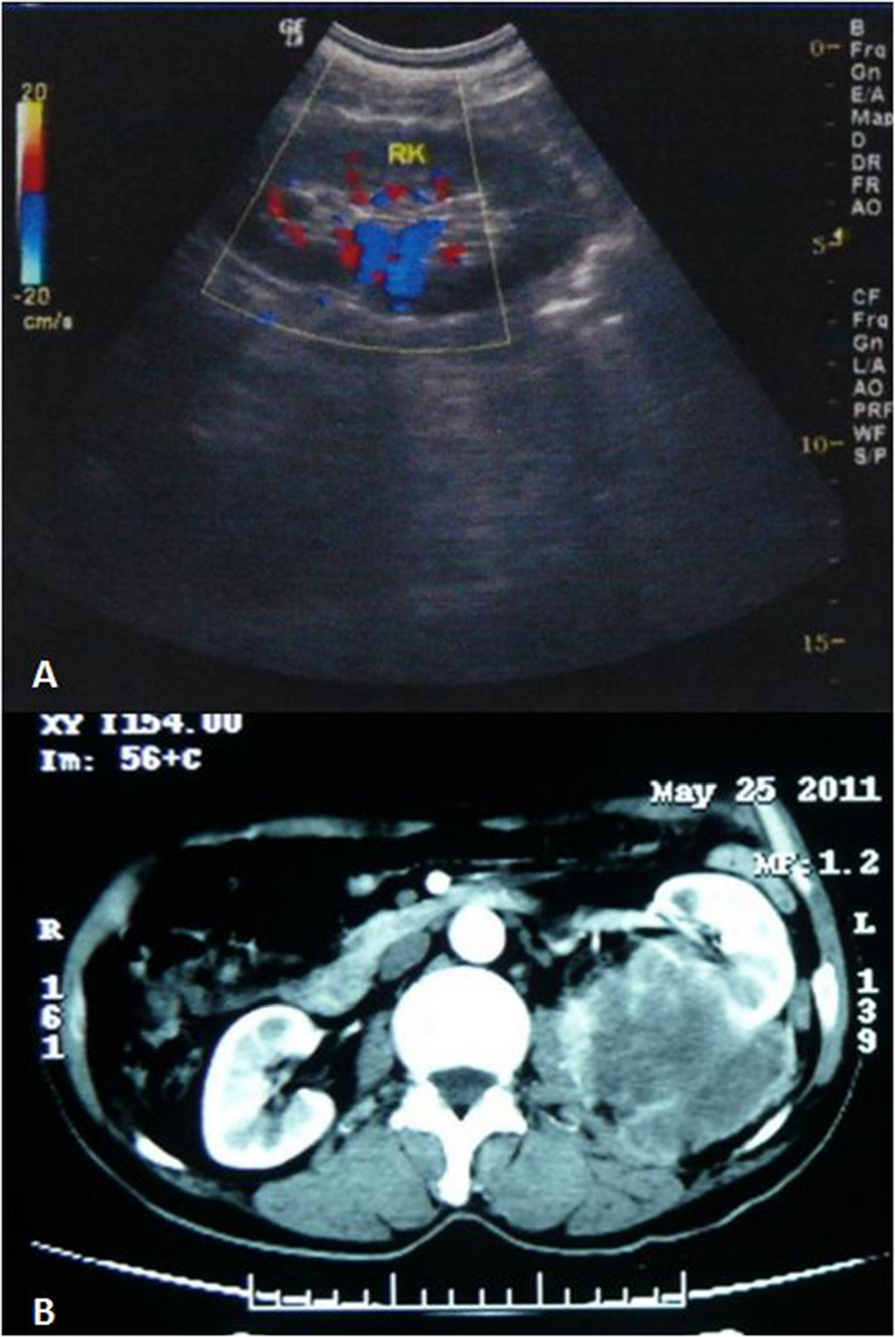
**Radiological features of the tumor. (A)** Ultrasound examination showed a space-occupying mass of low echogenicity within irregular margin. **(B)** Computed tomography showed a large mass in the left kidney.

A 69-year-old, previously healthy Chinese man underwent a routine physical examination three months ago, and a mass was discovered in his left kidney with ultrasound examination and computed tomography (Figure 1 A and B). Based on these findings, a clinical diagnosis of a malignant tumor of uncertain type with invasion of splenic hilum and adjacent peritoneum indicated a need for total left nephrectomy. The patient was taken up for surgery. Left kidney resection was performed subsequently as well as spleen and part of peritoneum.

**Figure 2:**
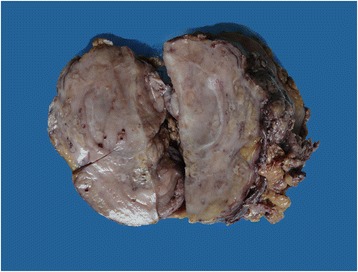
**Macroscopic features of the kidney.** There was an 11 cm x 8 cm grey white, hard solid mass in the renal parenchyma cross section without cysts.

Grossly, there was an 11 cm × 8 cm grey white, hard solid mass in the renal parenchyma cross section without cysts (Figure 2). Several hard nodes could be touched in adipose tissue of hilum of spleen and peritoneum. Histopathological examination of the mass showed a biphasic tumor composed of benign epithelium and malignant spindle shaped mesenchymal cells. Similar morphological features were found in the soft tissue in hilum of spleen and peritoneum.

The patient died of infection and multisysterm organ failure approximately two weeks after the surgery.

## Methods

Surgical specimen was fixed in 10% buffered neutral formalin and paraffin sections were stained with hematoxylin and eosin.

Immunostaining was performed by an enhancement method based on repetitive microwave heating of slides that were placed into 0.01 M citrate buffer at pH 6.0. A panel of antibodies (Table 1) was used. Binding of primary antibodies was visualized with an Envision two-step method. Diaminobenzidine was used as the chromogen. Nuclei were stained with Mayer’s hematoxylin. Appropriate positive and negative controls were included.

**Table 1:** **Antibodies and dilutions used in the evaluation of metanephric adenosarcoma of the kidney**

**Antibody**	**Dilution**	**Source**	**epithelium**	**stroma**
Vimentin	prediluted	Dako	-	+
CD10	1:20	Dako	-	+
AE1/AE3	1:20	Dako	+	-
CD57	1:50	Dako	+	-
WT-1	prediluted	Dako	-	−/+
EMA	1:100	Dako	+	-
CK7	1:200	Dako	+	-
CD117	1:50	Dako	+	-
CD56	1:100	Novocastra	-	-
Syn	1:100	Dako	-	-
CgA	1:400	Dako	-	-
CD34	1:50	Dako	-	-
α-inhibin	1:50	Dako	-	-
SMA	prediluted	Dako	-	-
Caldesmon	prediluted	Dako	-	-
MyoD1	1:200	Dako	-	-
Myogenin	1:50	Dako	-	-
S-100	prediluted	Dako	-	-
CD99	1:50	Dako	-	-
Ki67	1:40	Dako	<1%	60%

For fluorescence in situ hybridization (FISH), paraffin-embedded 5-μm sections were deparaffinized and the locus-specific probe (Vysis LSI SYT [18q11.2] Dual-Color, Break-Apart Rearrangement Probe) was used according to the manufacturer’s protocol. The fluorescence signals were analyzed using an Olympus BX51 fluorescence microscope (Olympus, Tokyo, Japan) equipped with appropriate filters and imaged using Vysis software. At least 100 cells were scored.

For BRAF mutation analysis, paraffin-embedded tissue samples were macrodissected to remove stromal contamination and to ensure tumor cellularity of ≥80%. Briefly, DNA extracted from the case was amplified and sequenced, using the BRAF Pyro Kit (QIAGEN) designed to detect mutations in codon 600. The kit was used according to the manufacturer’s instructions. GTCTCAGTCGTATGTAGTCTAG was used as the nucleotide dispensation order.

## Results

**Figure 3:**
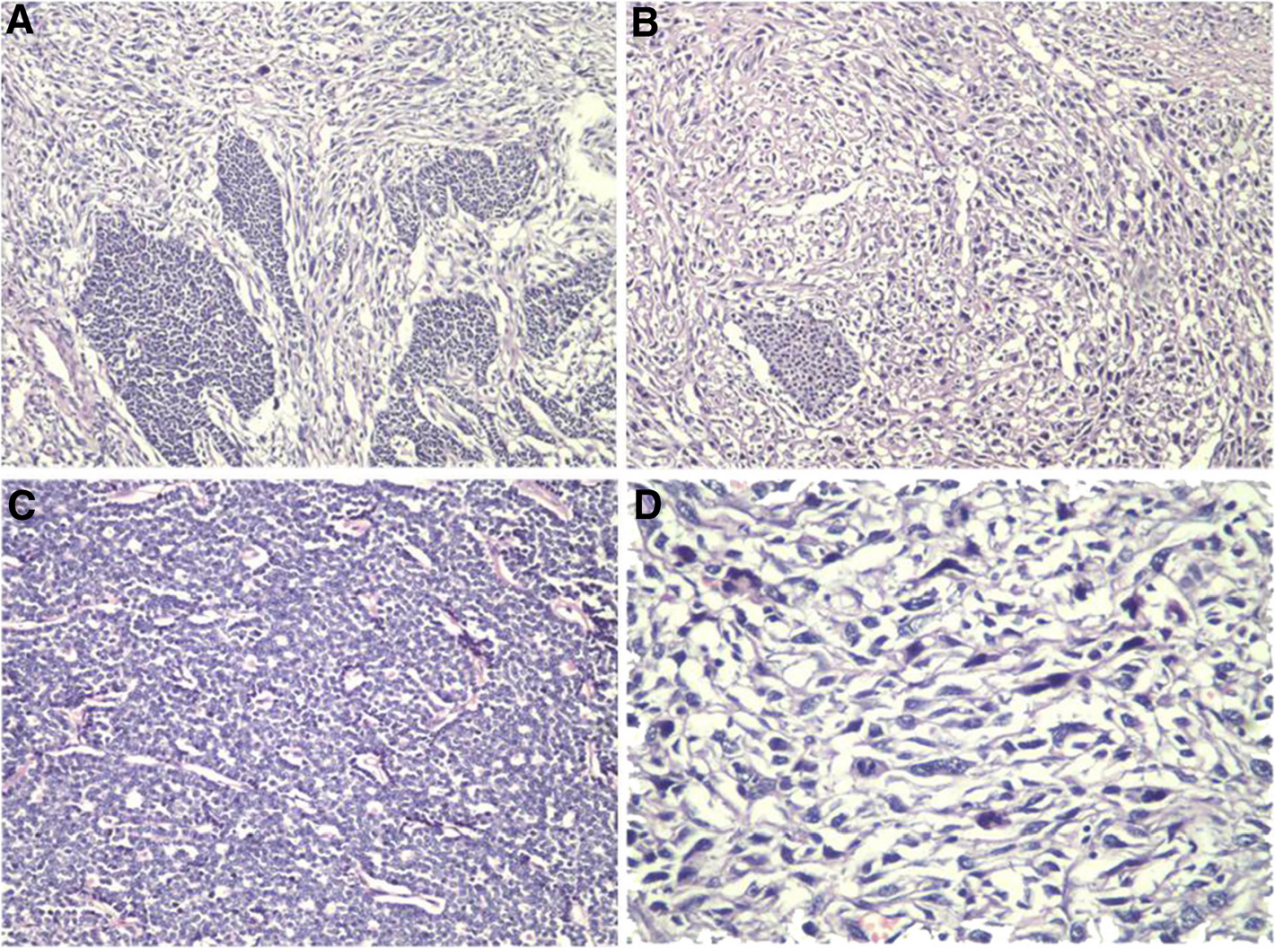
**Histopathological features of the tumor.** The unencapsulated tumor consisted of epithelium and stroma components. **(A)** Some areas were predominantly composed of epithelium identical to metanephric adenoma (H & E, ×200); **(B)** some areas were predominantly stromal, identical to high-grade sarcomatous tumor (H & E, ×200); **(C)** there were hypercellular uniform cells in solid-acini pattern, and the cells were small, round and tightly packed in MA area (H & E, ×400). **(D)** There were highly cellular, consisting of polymorphic spindle cells with obvious nuclear atypia and frequent mitosis in sarcomatous area (H & E, ×400).

Microscopically, the unencapsulated tumor consisted of epithelium and stroma components (Figure 3A and B). The relative proportion of stroma to epithelium in the sections varied over a wide range. The two components were frequently intimately associated with each other in the tumor with sharp border. Some areas were almost entirely composed of epithelium identical to MA (Figure 3C). Others were predominantly stromal, identical to high-grade sarcomatous tumor (Figure 3D).

In benign epithelium area, there were hypercellular uniform cells in solid-acini pattern, Papillary architecture could be seen focally, and no Ductal and glomeruloid structures could be seen. No psammoma bodies or osseous metaplasia were observed either. Tumors cells were small, round and tightly packed. Motitic figures were rare and absent. There were no atypical ones either. In malignant stroma area, there were highly cellular, consisting of polymorphic spindle cells with obvious nuclear atypia and frequent mitosis (16/10 high power fields).

**Figure 4:**
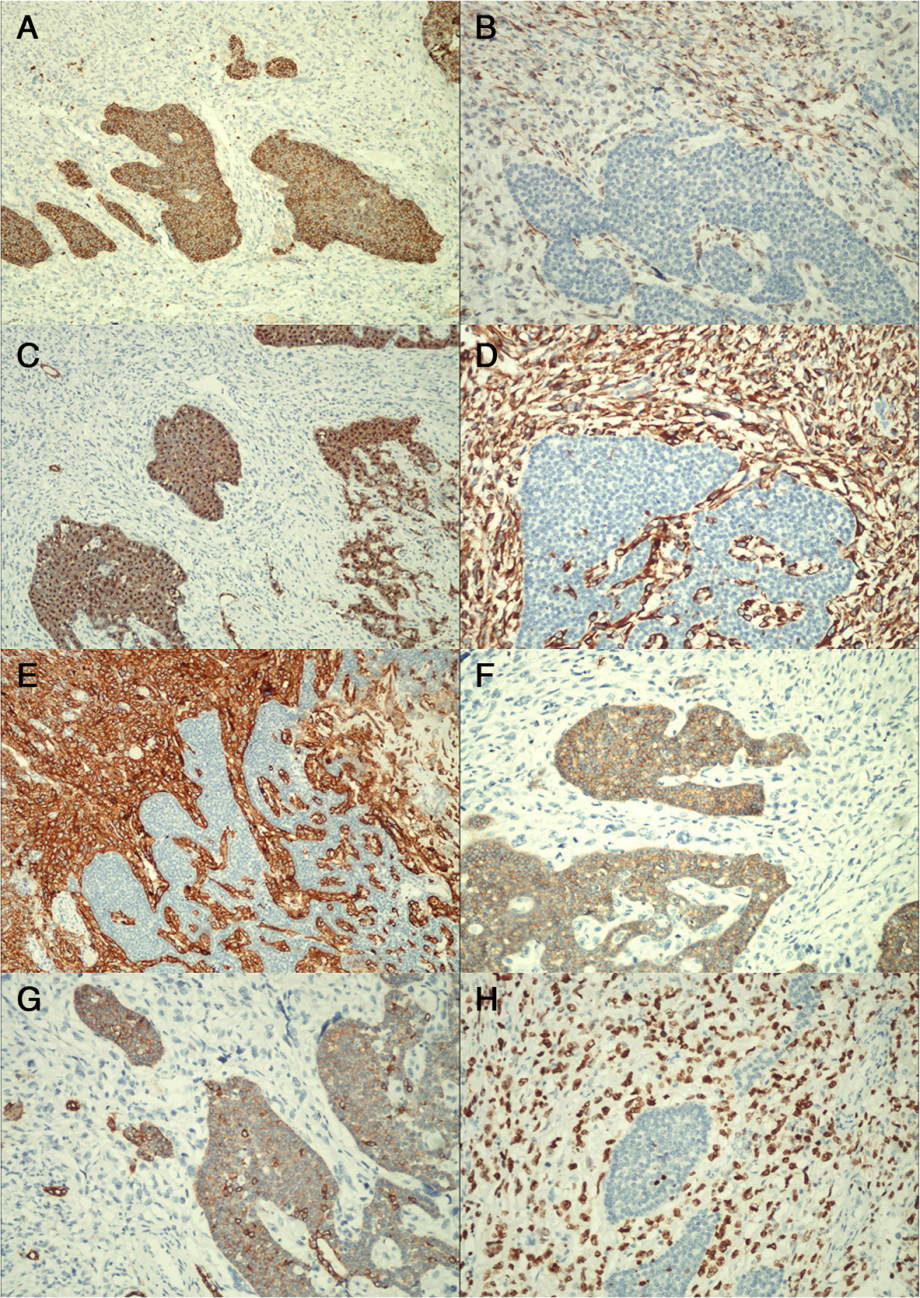
**Immunohistochemical findings of the biphasic tumor: (A)** **CD57;** **(B)** **WT1;** **(C)** **EMA;** **(D)** **Vimentin;** **(E)** **CD10;** **(F)** **CD117;** **(G)** **AE1/AE3;** **(H)** **Ki67.** (**A-H**, ×200).

Immunohistochemical results showed that the epithelial component was positive for CD57 (Figure 4A), AE1/AE3 (Figure 4G), cytokeratin (CK) 7, epithelial membrane antigen (EMA) (Figure 4C) and CD117 (Figure 4F); other markers including WT-1 (Figure 4B), CD10, CD56, synaptophysin (Syn), Chromogranin A (CgA), CD34 and α-inhibin were negative. The stromal spindle cells were positive for Vimentin (Figure 4D) and CD10 (Figure 4E), and scattered WT-1 staining (Figure 4B), but negative for smooth muscle actin (SMA), Caldesmon, Desmin, MyoD1, Myogenin, S-100, HMB45, MelanA, CD99 and TFE3. The Ki-67 labeling indices were up to 60% in sarcomatous element and <1% in epithelial element (Figure 4H), respectively.

FISH analysis for synovial sarcoma showed negative result since more than 90% of the counted cells didn’t show separated green and orange signals indicative of rearrangement of the *SYT* gene. Mutation analysis showed that no BRAF V600E mutations were detected in this case.

## Discussion

Metanephric neoplasms comprised a spectrum of kidney tumors containing renal epithelial or stromal cells or both [1,3]. The majority of cases are usually benign without atypical histological features in not only epithelial component but also stromal one; Metanephric adenoma (MA) is a common subtype in previous reports [4-6]. Rare malignant or metastatic diseases have been reported [7-9]. As we known by checking previous references, only one case of metanephric neoplasm with malignant stroma, named as “metanephric adenosarcoma”, was reported in 2001 [2]. Here we believe that this biphasic tumor we present is the second case of metanephric adenosarcoma.

Microscopically, the biphasic tumor has two components, one is epithelial element, which composed of solid or tightly packed small, monotonous, and round acini and focal papillary structures; tumor cells possessed scant cytoplasm, usually pale or light pink, with small uniform nuclei, delicate chromatin, and absent or inconspicuous nucleoli. These morphological features favor the diagnosis of MA. The epithelial component positive for CD57 also gave a valuable implication of MA although there was no immunohistochemical profile specific for metanephric neoplasia [10-12]. However, the epithelial component was negative for WT-1 immunohistochemically [10,13]. It was different from a majority of reports of MA with WT-1positive staining. According to Oligac’s report, 30% of MA cases he reviewed shown WT-1 negative staining because of different WT-1 antibodies application [12]. We believe that the histological features and immunohistochemical profile of epithelial components were identical to MA in our case.

To date, there is no special molecular pathology analysis used as diagnostic one for MA [14,15]. Recently Choueiri TK et al. confirmed BRAF V600E mutations were present in approximately 90% of all MA cases [16], serving as a potential valuable diagnostic tool in the differential diagnosis, but there was no BRAF mutation in our case.

The stromal element was composed of spindle cells with sarcomatous features. The cells were elongated with abundant cytoplasm and irregular nuclei, hyperchromatic chromatin, and plenty of atypical mitotic figures, and necrosis was not identified. The histological features of stroma were identical to high-grade sarcoma. Immunohistochemistry showed that the malignant stroma revealed no specific differentiation direction with Vimentin and CD10 positive staining, but others including SMA, Caldesmon, Desmin, MyoD1, Myogenin, S-100, HMB45, MelanA and CD99, were negative.

Because the mass was a morphologically biphasic tumor in kidney, we considered the following as differential diagnoses of this tumor base on gross and microscopic resemblance: mixed epithelial and stromal tumor (MEST) with malignant transformation, adult nephroblastoma, and renal synovial sarcoma (SS) and sarcomatoid renal cell carcinoma.

MEST is a rare adult renal neoplasm and microscopic analog of our case. The tumor is ordinary benign one, although some malignant transformation has been reported [17,18]. Our case differs from MEST with malignant transformation by the absence of multiple cysts macroscopically and microscopically and lack of hobnail appearance of the epithelial cells. A cystic renal tumor consisting of benign epithelial and malignant stromal components named “adenosarcoma” was reported recently, and the author believed that it was a novel entity [19]. We thought our case was a different one because it had no cystic change. Diagnosis of adult nephroblastoma could not be made because the tumor had no typical triphasic pattern morphologically and negative WT1 staining either. Primary synovial sarcoma (SS) in kidney is a rare distinct entity which is potentially a true biphasic tumor [20]. The epithelial component in current tumor lacked the cellular atypia associated with biphasic SS, and the stromal elements were highly pleomorphic with distinct nucleoli in contrast to the rather monomorphic spindle cells with inconspicuous nucleoli in typical SS. Immunohistochemistry results also contradicted the diagnosis of SS. FISH analysis showed no rearrangement of the *SYT* gene, which did not support the diagnosis of synovial sarcoma either. Sarcomatoid Renal Cell Carcinoma can be ruled out since there was no carcinomatous component and CD10-/CD57 + profile of epithelial element.

## Conclusions

In summary, we believe the macroscopic, histopathologic features of our case most closely resemble the metanephric adenosarcoma as M. M. Picken et al. named in 2001, and metanephric adenosarcoma should be in the expanded spectrum of metanephric neoplasia as a malignant stromal variant. The diagnosis of metanephric adenosarcoma, an extremely rare malignant renal tumor, required the incorporation of clinical information, histopathologic features, related markers IHC staining and molecular pathology analysis.

## Consent

Written informed consent was obtained from the patient for the publication of this report and any accompanying images.
